# Evaluating a systematic intensive therapy using continuous glucose monitoring and intermittent scanning glucose monitoring in clinical diabetes care: a protocol for a multi-center randomized clinical trial

**DOI:** 10.3389/fcdhc.2023.1247616

**Published:** 2023-10-18

**Authors:** Arndís F. Ólafsdóttir, Marcus Lind

**Affiliations:** ^1^ Department of Molecular and Clinical Medicine, Sahlgrenska Academy, University of Gothenburg, Gothenburg, Sweden; ^2^ Department of Medicine, NU Hospital Group, Uddevalla, Sweden; ^3^ Department of Medicine, Sahlgrenska University Hospital, Gothenburg, Sweden

**Keywords:** continuous glucose monitor (CGM), intermittent scanning -continuous glucose monitor (isCGM), telemedicine, diabetes nurse, type 1 diabetes, glycemic control

## Abstract

**Introduction:**

As many people with type 1 diabetes find it hard to reach the recommended glycemic goals, even with CGM, this study aims to determine if a closer, digitally supported collaboration on interpreting CGM data together with a diabetes nurse can improve glycemic control.

**Methods and analysis:**

A total of 120 individuals, 18 years and older and with HbA1c ≥ 58 mmol/mol will be included in the study at 8 different sites in Sweden and Norway. To be included, the participants must use a CGM or isCGM and be able to upload the data to the appropriate online service for their clinic and sensor. Both those with insulin pumps and insulin pens will be included in the study. Participants will be randomized into two different groups, that is, the intensive therapy group and the control group. The intensive therapy group will upload their glucose data weekly for the first 4 months and have telephone contact with their diabetes care team to receive support in interpreting CGM data and taking appropriate actions if their mean blood glucose level is above 8.4 mmol/L. After the 4-month-long intensive treatment phase, both randomized groups will have the same number of clinical visits and receive the same type of diabetes support.

**Discussion:**

It is of great importance to find new ways to help people with type 1 diabetes manage their condition as well as they can to help them achieve better glycemic control so that hopefully more people can achieve the recommended glycemic goals, which are associated with fewer diabetes complications. If it is shown that people with type 1 diabetes achieve better glycemic control with intensive therapy, then this can be incorporated into clinical praxis as an option for those not currently reaching the recommended glycemic goals.

**Clinical Trial Registration:**

https://clinicaltrials.gov/study/NCT03474393?locStr=Uddevalla,%20Sweden&country=Sweden&distance=50&cond=Diabetes&aggFilters=ages:adult%20older&state=V%C3%A4stra%20G%C3%B6taland%20County&city=Uddevalla&page=4&rank=34, identifier 03474393.

## 1 Introduction

Good glycemic control is a key element in the reduction of long-term diabetes complications in people with type 1 diabetes (1). Over the last few years, continuous glucose monitors (CGMs) have been shown to be an efficient way of improving glycemic control, both in people treated with insulin pumps and those treated with multiple daily insulin injections (2, 3). In addition, it was shown that CGM led to an improvement of time in hypoglycemia as well as quality of life and their levels of hypoglycemic confidence (4). Intermittent scanning CGMs (isCGMs) have also recently been shown to efficiently reduce time in hypoglycemia (5, 6).

A CGM is a subcutaneous sensor that continuously estimates blood glucose levels, which are then displayed on a small handheld monitor or a mobile phone. A CGM also informs users of glucose trends and alerts them if their blood glucose levels are low or high (7). An isCGM is a subcutaneous sensor that is placed in the upper arm and needs to be scanned with a small handheld monitor or by a mobile phone to measure estimated blood glucose levels and to show the current glucose trends (8).

Although CGMs and isCGMs are used by approximately 90% of patients with type 1 diabetes in Sweden, 70% of patients still do not achieve the good glycemic control associated with a low risk of diabetes complications (9). This is in line with findings in clinical trials, which demonstrate that although the use of CGMs can efficiently lower HbA1c levels, a reduction of only approximately 0.4% has been demonstrated, meaning that the use of CGMs alone is insufficient for solving the problem of poor glycemic control for most patients (3, 10, 11).

However, in earlier studies, patients have in general received education about their systems only at the beginning of the studies and then gone on to use them in their daily life, with regular clinical visits as support. It is possible that the effects of isCGM and CGM on glycemic control could be greatly improved with greater clinical support. A CGM/isCGM could even be used as a motivational tool for patients that increases communication between them and their caregivers. It is possible that diabetes care could be developed into forms of assistance for patients other than the current system of regular clinical visits every 3–6 months.

isCGM and CGM data can be electronically transferred to the caregiver. This opens new opportunities for more intense discussion and support of glucose values and trends that are closer to actual daily living. The question is whether or not such an approach would be more beneficial than the current approach of regular clinical visits and what effects could be obtained. If mean blood glucose levels are elevated, glucose data could be transferred weekly to the diabetes care team for guidance. In addition to enabling patients to obtain assistance, such an approach could possibly improve patient motivation. Specific individual targets could be set for each patient, depending on their individual needs and how far from the recommended guidelines their measurements lie. In this way, they could achieve several sub-goals in the process of achieving their final goal.

Currently, diabetes care teams in many countries are not financially set up to provide support *via* telephone contact or other media. However, if distance contact was shown to be more effective, it is likely that financial resources would be allocated to the provision of this service and that the systems for registering the financial costs associated with such work could be changed.

The aim of the study is to evaluate whether glycemic control in persons with type 1 diabetes can be improved by a close collaboration between persons with diabetes and diabetes-care teams using isCGM and CGM data. In addition, we will evaluate if this approach has a sustained effect on glycemic control after it is discontinued.

The primary objective of this study is to evaluate whether or not systematic intensive therapy in combination with transferring isCGM and CGM data to diabetes care teams will improve glycemic control (measured by HbA1c levels at baseline and after 18 weeks) compared with conventional care in people with type 1 diabetes with impaired glycemic control over an 18-week period.

The secondary objective is the comparison of the following variables between patients with type 1 diabetes who have been randomized into the systematic intensive therapy group and the conventional care group:

- HbA1c levels at 32 weeks;
- HbA1c levels at 52 weeks;
- Mean blood glucose levels at 18, 32, and 52 weeks;
- Change in time in range (3,9-10 mmol/l) and time in target (3,9-7,8mmol/l) at week 18;
- Change in time in range (3,9-10 mmol/l) and time in target (3,9-7,8mmol/l) at week 52;
- Glycemic variability as measured by standard deviation, CV, and MAGE at 18, 32, and 52 weeks;
- Time in hypoglycemia at 18, 32, and 52 weeks;
- Time in hyperglycemia 18, 32, and 52 weeks;
- Hypoglycemia confidence (hypoglycemia confidence scale) at 18, 32, and 52 weeks;
- Diabetes distress (DDS) questionnaire results at 18, 32, and 52 weeks; and
- Treatment satisfaction (measured by DTSQs and DTSQc questionnaire results) at 18, 32, and 52 weeks.

## 2 Method and analysis

### 2.1 Study design and locations

**Figure 1:**
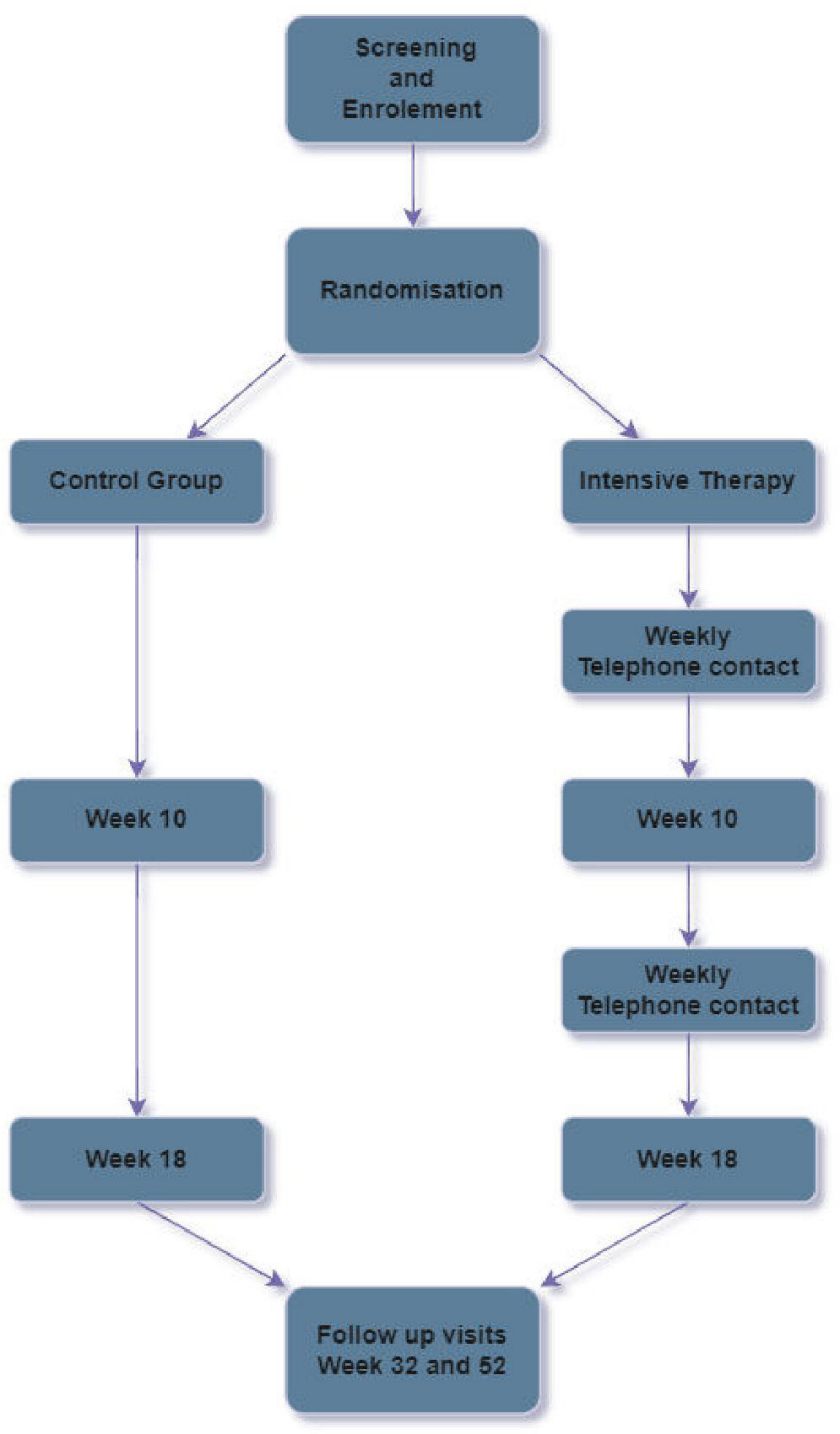
Flowchart of study.

The study will take place at eight outpatient clinics in Sweden and Norway. It is a non-blinded, multi-center randomized clinical trial with a parallel design that we plan to conduct between November 2019 and March 2023. The study will include 120 individuals who will be randomized into two equal groups, that is, the control group and the intensive therapy group. Intensive therapy will be conducted over 18 weeks, followed by a 34-week follow-up period. The study design is shown in **Figure 1**.

### 2.2 Eligibility criteria

Patients with type 1 diabetes with HbA1c levels of ≥ 58 mmol/mol, who are currently using a CGM or an isCGM and who have the ability to be able to upload data from their devices to the appropriate online service for their clinic and sensor from home, will be included. The full list of inclusion and exclusion criteria is shown in **Table 1**.

**Table 1:** Inclusion/exclusion criteria.

Inclusion criteria: • Informed consent obtained before trial-related activities (i.e., any activity that would not have been performed during routine patient management); • Clinical diagnosis of type 1 diabetes; • Adult patients over 18 years of age; • HbA1c level ≥ 58 mmol/mol; • Currently using a CGM or isCGM; • Is able to upload and share isCGM/CGM data. Exclusion criteria: • Type 2 diabetes; • Diabetes duration <1 year; • Long-term systemic glucocorticoid treatment during the last 3 months; • Planning to change or have changed diabetes treatment in the last 3 months, regarding change from MDI to insulin pump or started/stopped CGM or IsCGM • Current or planned pregnancy or breastfeeding during the next 12 months; • Planned move during the next 12 months making it impossible to participate in study activities; • Other reason determined by the investigator to not be appropriate for participation.

### 2.3 Randomization

Patients will be allocated 1:1 to systematic intensive therapy or conventional therapy using a minimization algorithm to achieve balance between treatment groups on important prognostic factors, that is, age, sex, HbA1c level, treatment type (injections or pump), and sensor type (CGM/isCGM). A centralized data system will be used. The use of this randomization method will increase the probability that key variables overall are evenly distributed between treatment groups (12–14).

### 2.4 Treatment

#### 2.4.1 Systematic intensive therapy

Patients randomized to the systematic intensive therapy group will continue to follow their regular planned clinical visits and contacts. As part of the patient-centered care provided, all patients will be taught how to upload CGM/isCGM data to their home computer/laptop. In addition, by drawing on their prior knowledge of how to use the software suitable for their device, they will be taught to interpret the data. They will be taught how to interpret the data by looking at variables such as:

A) High/low overnight and morning profile;

B) Excursions before and after meals;

Timing of insulin in relation to mealtimes and exercise; and

C) Time spent in various glycemic ranges and glycemic variability *via* the standard deviation.

D) For most devices, it will be possible to use Diasend^®^, a software that is compatible with most CGM systems, but for some CGM systems specific software will be needed, such as CareLink™ for Medtronic products or LibreView for Libres isCGMs.

We will explain the relationship between mean glycemic control and HbA1c levels to the patients, and we will also give them a graph representing this relationship that further explains how these two variables are connected.

An individual HbA1c-level goal will be discussed with patients, and a target for mean blood glucose levels that matches this HbA1c-level goal will be set. The mean glucose goal will be discussed in relation to the mean blood glucose level at randomization. The patients will be taught how to upload data from their device during the first visit and will receive further assistance if necessary. If needed, more support will be given in the beginning by internet or telephone contact as patients upload data from their device at home.

We intend that the first contact *via* telephone will take place up to and no later than 1 week after randomization. The patient will be expected to have uploaded data from their device prior to this contact so that both the caregiver and patient have access to all CGM glucose profiles for the previous week. The visit will take place the same day each week ±1 day.

Together with the participant, the caregiver will conduct an analysis of the prior week’s glucose profiles. This analysis will be conducted in relation to mean blood glucose levels and the standard deviation, before-bedtime data, the overnight glucose profile, blood glucose levels before and after meals and exercise, and time spent in hypoglycemia. The patient will also be able to discuss any particular situations that have proven to be more difficult than others.

Using their unique expertise, the caregiver will be expected to make an overall judgement regarding what needs to be done to improve the patient’s glucose profiles.

If the patient has achieved their first mean blood glucose goal but not the recommended goal of 8.4mmol/L, a new target will be decided on. The recommended mean blood glucose level of 8.4mmol/L corresponds to an estimated HbA1c level of 52 mmol/mol, which is the national recommendation in Sweden (15). Therefore, if patients achieve a mean blood glucose level of 8.4mmol/L, they are expected to have an HbA1c level that is associated with a lower risk of complications (16).

During the first 4 weeks, weekly contact by telephone will be made. After this time, it might be necessary to reduce this to every second week, depending on the patients’ mean blood glucose levels.

If the patient achieves the recommended blood glucose goal of mean glucose < 8.4 mmol/L, no telephone contact will be made that week, but the participant will need to upload their data again the following week, and if their mean blood glucose level is found to be above 8.4 mmol/L, contact *via* telephone will need to be made again.

Clinical visits will be scheduled after 10, 18, 32, and 52 weeks for both groups to evaluate the effects of each care provision model on the patients’ HbA1c levels and other glycemic variables (mean blood glucose levels, SD of blood glucose levels, time in hypoglycemia and hyperglycemia, and time in range).

#### 2.4.2 Conventional therapy

Clinical visits will be scheduled 10, 18, 32, and 52 weeks after randomization for HbA1c level measurement and downloading of CGM/isCGM curves. During weeks 18, 32, and 52, the participants will fill in follow-up questionnaires from those registered at baseline. The visits at weeks 10 and 18 will take place exactly 10 and 18 weeks after randomization (± 1 week), and the visits at weeks 32 and 52 will take place exactly 32 and 52 weeks after randomization (± 2 weeks).

#### 2.4.3 Follow-up phase

After 18 weeks, the participants will return to their regular care schedule at their diabetes clinic, but HbA1c levels will be measured at 32 and 52 weeks. No distance intervention will be carried out during this time, but glucose data will be uploaded from their devices at each visit. The participants will be encouraged to, and hopefully will continue to, upload data from their devices at home so that they can analyze their glucose profiles as they have been taught during the intervention. If they actively make contact with their diabetes team due to technical problems regarding uploading data from their devices or with specific questions regarding their analysis, advice and support will be given, but no further contact will be planned.

#### 2.4.4 Data collection

In addition to randomization, the patients will have visits at weeks 10,18, 32, and 52 with a diabetes nurse. At the week 10 visit with the diabetes nurse, data from the CGM/isCGM will be uploaded, and HbA1c levels will be measured. At the week 18, 32, and 52 visits, data from the CGM/isCGM will be uploaded, and the following variables will be measured: HbA1c level, weight, type of insulin and doses of insulin, AEs, SAEs, DTSQs score, DTSQc score, DDS score, and hypoglycemia confidence score. At week 52, a physical examination will be conducted. Detailed trial procedures are shown in **Table 2**.

**Table 2:** Trial procedures.

Variables		Visits **				
	Inclusion (visit 1)*	Randomization (visit 2)	10-week follow-up visit 3	18-week follow-up (visit 4)	32-week follow-up (visit 5)	52-week follow-up (visit 6)
Visit window		Scheduled within 28 days after inclusion	± 1 weeks	± 1 weeks	± 2 weeks	± 2 weeks
Informed consent	X					
Inclusion/exclusion criteria	X					
Demographics, medical history	X					
Physical examination	X					X
HbA1c level	X	X	X	X	X	X
Uploading device	X	X	X	X	X	X
Education on uploading device		X				
Weight		X		X	X	X
DTSQs		X		X	X	X
DTSQc				X		
DDS scale		X		X	X	X
Hypoglycemia confidence scale		X		X	X	X
AEs (severe hypoglycemia and diabetes ketoacidosis) and SAEs		X	X	X	X	X

* Before the visits in the schedule above, patient information will be given either via telephone or at a clinical visit.

** If randomized to systematic intensive treatment, the first telephone contact will take place 1 week after randomization and, after that, on a weekly basis or until mean blood glucose levels reach the target level.

### 2.5 Endpoints

The primary endpoint will be the change in HbA1c level from baseline to week 18. All predefined endpoints are shown in **Table 3**.

**Table 3:** predefined endpoints.

The primary endpoint will be the change in HbA1c level from baseline to week 18. The secondary endpoints will be: • Change in HbA1c levels from baseline to week 32; • Change in HbA1c levels from baseline to week 52; • Change in time in range (3,9-10mmol/l) and time in target (3,9-7,8mmol/l) from baseline to week 18; • Change in time in range (3,9-10mmol/l) and time in target (3,9-7,8mmol/l) from baseline to week 52; • Change in mean blood glucose levels from baseline to 18, 32, and 52 weeks; • Change in glycemic variability, measured by standard deviation, CV, and MAGE from baseline to 18, 32, and 52 weeks; • Change in time in hypoglycemia from baseline to 18, 32, and 52 weeks; • Change in time in hyperglycemia from baseline to 18, 32, and 52 weeks; • Change in hypoglycemic confidence score from baseline to 18, 32, and 52 weeks; • Change in DDS score from baseline to 18, 32, and 52 weeks; • Change in DTSQs score from baseline to 18, 32, and 52 weeks and DTSQc score at 18, 32, and 52 weeks.

### 2.6 Monitoring and laboratory analyses

Researchers at Wallenberg Laboratory at the University of Gothenburg will monitor the trial. Capillary tests will be carried out at each visit using a DCA Advance analyzer, which is Equalis calibrated.

### 2.7 Statistics

All analyses will be specified in the statistical analysis plan (SAP) prior to database lock.

#### 2.7.1 Sample size calculation

The study will be designed to detect if there is an improvement of 0.4% in patients’ HbA1c levels from baseline to the 18-week follow-up visit. An SD of 0.8% for change in HbA1c level has been assumed for both treatment groups, indicating that 54 individuals per group are needed to obtain a power of 80% at an alpha level of 0.05. Accounting for a dropout rate of 10%, 120 individuals will be needed. Initially, the plan was to include 142 individuals, which would also allow the detection of a 0.4% improvement in HbA1c levels. However, due to the difficulty in recruiting during the COVID-19 pandemic, it was decided to reduce this to 120 individuals. The protocol amendment was made and approved by the ethics committee in June 2022.

#### 2.7.2 Primary and secondary analyses

The primary analysis will be of the change in HbA1c levels from baseline to the 18-week follow-up visit between the two treatment groups using analysis of covariance (ANCOVA), with the HbA1c level at baseline as a covariate on the ITT population using a two-sided test and with a significance level of 0.05.

Secondary analyses will be of:

- Change in HbA1c levels, mean blood glucose levels, glycemic variability and times in hypoglycemia and hyperglycemia, time in range, and time in target, analyzed in a similar way to those described above for the primary variable;
- Change in the DDS score and hypoglycemia confidence score from baseline to weeks 18, 32, and 52 between the two treatment groups using ANCOVA with the score of the evaluated variable at baseline as a covariate;
- The difference in DTSQc scores at week 18 between the two treatment groups using ANCOVA in case the assumption of normal distribution is met or, if it is not, by using a Mann–Whitney U-test.
- The difference in DTSQs scores at weeks 18, 32, and 52 between the two treatment groups using ANCOVA in case the assumption of normal distribution is met or, if it is not, by using a Mann–Whitney U-test.

## 3 Discussion

This is a description of the protocol for a randomized multi-center study that investigates the effect of intensive telephone contact to support the interpretation of CGM data and the taking of appropriate actions as a complement to traditional diabetes care, both in the short term and over a longer period.

In recent years, new continuous glucose monitoring and more advanced insulin pumps have been introduced to the market, e.g., HCL pumps, which have shown great glycemic improvement (17, 18). The current study will include only patients taking multiple daily insulin injections, using insulin pumps not connected to a CGM, or using sensor-augmented pumps where the basal rate stops if blood glucose levels are expected to fall below a certain level. However, it is essential to note that, currently, advanced insulin pumps are only used by a small minority of people with type 1 diabetes, although this number is expected to increase. From an international perspective, most type 1 diabetes patients do not even have the option of using an CGM/isCGM (19). For example, in Asia, Africa, South America, and Eastern Europe, capillary testing is still the most common glucose monitoring method and injections are still the most common method of insulin delivery. Hence, an understanding of how to best use CGM for patients using MDIs and simpler insulin pumps is knowledge that will remain essential for a long time. Moreover, patients with more advanced insulin pumps may also need more intensive counseling, and the current study will also indicate if such an approach could be of use for these patients. As technology improves, it is important to facilitate more person-centered care, and telemedicine can be one means of doing this (20).

Many patients have the possibility of using both CGM and isCGM, but of those, there are still too few who are achieving the recommended glycemic goals at a level that is sufficient to minimize diabetes complications. It is of great importance to gain knowledge of the potential benefits of systematic intensive therapy administered by a diabetes nurse in type 1 diabetes care. If the treatment improves glycemic control, diabetes distress, or hypoglycemic confidence, it could be a complement to the routine care provided by diabetes teams. If more people can achieve the recommended glycemic goals, their risk of diabetes complications will decrease. Furthermore, if it is shown that people with type 1 diabetes achieve better glycemic control with intensive therapy, this can be incorporated into diabetes guidelines as an option for those not currently achieving the recommended glycemic goals.

## Ethics statement

The studies involving humans were approved by Ethics Committee at the University of Gothenburg, Gothenburg, Sweden. The studies were conducted in accordance with the local legislation and institutional requirements. The participants provided their written informed consent to participate in this study.

## Author contributions

AÓ and ML designed the study. ML is the PI of the study. AÓ drafted the manuscript. Both authors reviewed and approved the final manuscript.
